# HIV-Related Myocardial Fibrosis: Inflammatory Hypothesis and Crucial Role of Immune Cells Dysregulation

**DOI:** 10.3390/cells11182825

**Published:** 2022-09-09

**Authors:** Eman Teer, Leanne Dominick, Nyasha C. Mukonowenzou, M. Faadiel Essop

**Affiliations:** 1 Centre for Cardio-Metabolic Research in Africa, Department of Physiological Sciences, Stellenbosch University, Stellenbosch 7600, South Africa; 2 Centre for Cardio-Metabolic Research in Africa, Division of Medical Physiology, Faculty of Medicine and Health Sciences, Stellenbosch University, Cape Town 8000, South Africa

**Keywords:** HIV, myocardial fibrosis, platelets, chronic inflammation, sudden cardiac death, heart failure, cardiovascular diseases

## Abstract

Although the underlying mechanisms driving human immunodeficiency virus (HIV)-mediated cardiovascular diseases (CVD) onset and progression remain unclear, the role of chronic immune activation as a significant mediator is increasingly being highlighted. Chronic inflammation is a characteristic feature of CVD and considered a contributor to diastolic dysfunction, heart failure, and sudden cardiac death. This can trigger downstream effects that result in the increased release of pro-coagulant, pro-fibrotic, and pro-inflammatory cytokines. Subsequently, this can lead to an enhanced thrombotic state (by platelet activation), endothelial dysfunction, and myocardial fibrosis. Of note, recent studies have revealed that myocardial fibrosis is emerging as a mediator of HIV-related CVD. Together, such factors can eventually result in systolic and diastolic dysfunction, and an increased risk for CVD. In light of this, the current review article will focus on (a) the contributions of a chronic inflammatory state and persistent immune activation, and (b) the role of immune cells (mainly platelets) and cardiac fibrosis in terms of HIV-related CVD onset/progression. It is our opinion that such a focus may lead to the development of promising therapeutic targets for the treatment and management of CVD in HIV-positive patients.

## 1. Introduction

There are currently ~38.4 million human immunodeficiency virus (HIV)-infected individuals globally, with ~28.7 million receiving combination antiretroviral therapy (cART) [1]. Increased access to cART has significantly improved the lifespan of people living with HIV (PLHIV). It has also attenuated viral replication and ensured a relatively well-maintained immune system, together with lowered opportunistic infections and associated mortalities [2]. The main cause of death in PLHIV has therefore shifted from acquired immunodeficiency disease (AIDS)-related immunocompromised states to non-AIDS age-related complications, such as cardiovascular diseases (CVD). In support, the proportion of global deaths due to CVD in PLHIV has increased from 2.5% to 4.6% during the past decade [3,4]. Although HIV-positive patients present with a variety of heart and vascular co-morbidities, certain cardiac disorders manifest with a greater frequency and display geographic variations. For example, in developed countries, PLHIV usually present with metabolic syndrome, hypertension, coronary artery disease, and atherosclerosis [4,5,6]. In contrast, complications such as hypotension, heart failure (HF)/sudden cardiac death (due to HIV-associated cardiomyopathy), and tuberculosis-associated pericarditis are far more prevalent in the sub-Saharan African region [7,8,9,10].

Despite this burgeoning health threat, the underlying mechanisms driving HIV-mediated CVD onset are still being elucidated. Of note, the role of chronic immune activation (despite cART) as a significant mediator in HIV-mediated CVD onset and progression is increasingly being highlighted [11,12]. In agreement, our laboratory recently found a strong interplay between immune activation, coagulation, and lipid subclass alterations in South African HIV-positive patients [9]. This data also revealed a robust negative correlation between either immune activation or coagulation, and diastolic blood pressure [9]. Chronic inflammation is a characteristic feature of various CVD and is regarded as a key contributor to diastolic dysfunction, HF, and sudden cardiac death [13]. HIV-mediated immune dysregulation can trigger downstream effects that lead to an enhanced release of pro-coagulant, pro-fibrotic, and pro-inflammatory cytokines [14]. This can subsequently result in an increased thrombotic state, endothelial dysfunction, and myocardial fibrosis [15,16]. The culmination of the interplay of such mediators can eventually lead to systolic and diastolic dysfunction and an increased risk for CVD.

While the pathogenesis of HIV-mediated CVD onset and progression is multi-factorial in nature, myocardial fibrosis is emerging as a key mediator underlying the manifestation of systolic and diastolic dysfunction [17]. In support, research findings have revealed prognostic associations between diffuse myocardial fibrosis and left ventricular (LV) remodeling in PLHIV [18,19]. Here, they found the manifestation of HIV-related myocardial fibrosis, especially in African women [19]. Such data support the development of more personalized approaches to screening and earlier interventions, to thereby help lower the burden of HF in PLHIV, especially in the sub-Saharan African region [20]. Furthermore, understanding the pathogenesis may help identify promising therapeutic targets. Considering this, the current review article will focus on (a) the contributions of a chronic inflammatory state and persistent immune activation, and (b) the role of immune cells (mainly platelets) and cardiac fibrosis, in terms of HIV-related CVD onset/progression, with an emphasis on HF and sudden cardiac death.

## 2. HIV Treatment and Cardiovascular Complications

Prior to cART, CVD manifestations of HIV infection included myocardial and peripheral disease, due to the direct effects of HIV, coinfections, and concomitant chronic inflammation [21]. The introduction of cART improved lifespans by viral load reduction and immune system restoration, but also came with side-effects, due to drug-toxicity and metabolic changes (e.g., dyslipidemia, altered glucose handling) [22,23,24]. Furthermore, there are variations between different antiretroviral classes and divergent responses within drug class types [23]. Older generation protein inhibitors, such as lopinavir/ritonavir, and nucleoside reverse transcription inhibitors, such as abacavir, stavudine and zidovudine, can induce dyslipidemia to increase CVD risk [24,25,26,27]. Moreover, body fat distribution changes are still evident years after cessation of antiretroviral use [28,29]. Earlier work, therefore, reported the occurrence of early-onset and aggressive coronary artery disease in PLHIV compared to uninfected individuals [30]. Currently, integrase inhibitors and C-C chemokine receptor 5 antagonists have replaced protease inhibitors as the first line therapy and appear to elicit negligible CVD toxicity, although there are concerns regarding the weight gain associated with their use and hence the need to assess their long-term effects in this context [31].

cART-treated HIV is associated with an increased incidence of myocardial fibrosis [32], as well as both systolic and diastolic LV dysfunction [33], and an up to two-times higher risk of HF [34]. Results from the Veterans Aging Cohort Study [35] showed that this manifests in various forms, such as HF with preserved ejection fraction, borderline HF with preserved ejection fraction, and those with a reduced ejection fraction. Furthermore, the occurrence of such HF subtypes occurs at an earlier stage in the PLHIV population versus uninfected individuals. However, the direct relationship between myocardial inflammation and fibrosis in HIV has been less well studied [13]. For PLHIV who are virally suppressed on cART, the risk of sudden cardiac death levels out to the risk observed in the general population [36]. Moreover, a Taiwanese study found that no specific cART class was associated with increased HF risk [37]. Meanwhile, a relatively small US study on virally suppressed women living with HIV (on integrase strand transfer inhibitors and nucleoside reverse transcription inhibitors) showed increased myocardial fibrosis and lowered diastolic function compared to HIV-negative women [38].

## 3. Immune Activation and Chronic Inflammation

HIV infection activates the innate and adaptive immune systems, which can result in a state of chronic infection that forms the basis of ongoing immune activation and immunodeficiency [14]. Inflammation is crucial in resolving infections, tissue damage, and maintaining a state of hemostasis [39]. While some degree of immune cell activation is essential to promote suitable responses to injury and activation of tissue repair processes, uncontrolled activation may lead to excess fibrosis and offset its beneficial effects [40].

The innate immune system consists of granulocytes (neutrophils, basophils, eosinophils), mast cells, and antigen presenting cells (macrophages and dendritic cells) [41]. Pathogen-associated molecular patterns and damage-associated molecular patterns can bind to cell surface toll-like receptors, which subsequently results in their activation [14,42]. The activated cells of the innate immune response produce pro-inflammatory cytokines, to further amplify the inflammatory response [39]. The acute inflammatory response starts rapidly, becomes more severe over short periods of time, and usually lasts for a few days [39].

However, if the pathogen-induced stimulation persists, the inflammatory process then acquires new characteristics that are more typically associated with chronic inflammation [39]. This is a slow, long-term state of inflammation that can last for prolonged periods and is induced by cytokines such as interferon-gamma (IFN-γ) that can promote activation of the adaptive immune system [42]. Here, T-cells play a significant role and differentiate into either CD4 (helping to orchestrate immune responses) or CD8 (destroying infected cells) cells [14,42]. Such cells, together with macrophages and natural killer cells, are key players for cell-mediated immunity, while B-cells produce antibodies and are responsible for humoral immunity [41]. Thus, the inflammatory response is the result of a complex interplay between multiple immune cells in the body.

Persistent immune activation and chronic inflammation occur during HIV-infection, despite cART adherence and suppressed viremia [42]. Chronic and persistent CD8^+^ T-cell activation (the marker of immune activation) rests on three important factors: (1) the persistent detection of HIV-specific effector cytotoxic T cells, (2) the presence of cell surface protein receptors that differentiate naïve T cells into differentiated effector phenotypes [43], and (3) an acute/active cytokine profile detected in serum. Ongoing immune activation and resulting inflammation can lead to immune-related perturbations [44]. Moreover, circulating monocytes and tissue macrophages are both susceptible targets of HIV-1 infection, and the early host response determines whether the infection becomes pathogenic or not. For example, monocytes and macrophages can contribute to the HIV reservoir (and viral persistence) and influence the initiation/extension of immune activation and chronic inflammation, despite cART [45]. Here, the inflammatory response is attenuated (when not required) and becomes chronic if there is a persistent source of activation and/or due to defective control mechanisms [46]. The harmful consequences of persistent immune activation and inflammation during HIV-infection have been extensively reviewed in the previously published literature [13,46,47].

## 4. Persistent Immune Activation, Chronic Inflammation, and Cardiac Fibrosis

**Figure 1:**
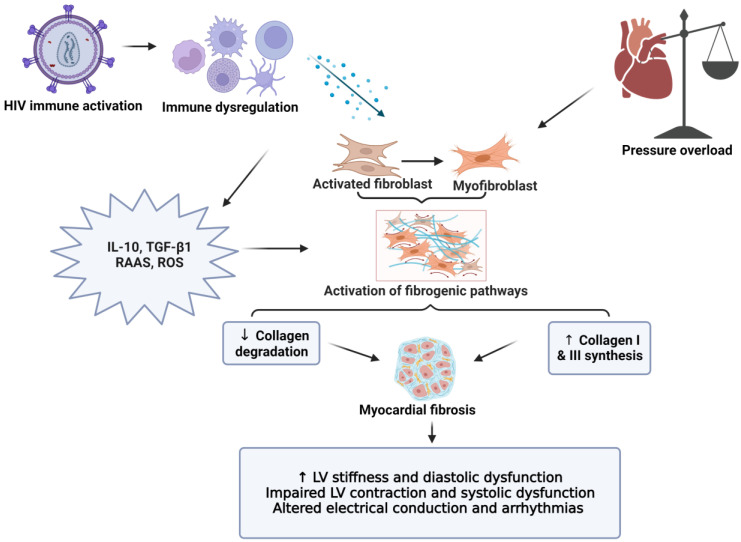
The role of myocardial fibrosis in CVD pathogenesis. The inflammatory hypothesis is considered a main driver of CVD complications in HIV-positive individuals. Persistent immune activation leads to a chronic inflammatory state that includes relatively high levels of inflammatory and pro-fibrotic cytokines (IL-10, TGF-β), together with RAAS activation. This subsequently enhances pro-fibrotic pathways (increased collagen I and III deposition), while also lowering collagen degradation. The increased collagen leads to LV stiffness and diastolic dysfunction (early sign of myocardial fibrosis). Myocardial fibrosis is a contributor to diastolic and systolic dysfunction, HF, and sudden cardiac death. LV: left ventricle, RAAS: renin-angiotensin aldosterone system, ROS: reactive oxygen species, IL: interleukin, and TGF: transforming growth factor.

Chronic inflammation and immune dysfunction increase the risk of cardiovascular morbidities and mortalities through endothelial dysfunction, hypercoagulation, and myocardial fibrosis [42,48,49,50,51]. The persistent activation of the innate and adaptive immune systems (monocytes/macrophages and T cells, respectively) results in increased circulating pro-inflammatory and pro-fibrotic cytokines (Figure 1) [12,49,52,53,54,55]. Higher circulating cytokine levels can contribute to hypercoagulation, endothelial dysfunction, and fibrotic remodeling, which increase the risk of CVD onset in PLHIV [32,50,56,57,58,59]. Fibrotic remodeling due to immune dysfunction is an important area of research, due to its detrimental effects on cardiac function, and its links to HF and sudden cardiac death in PLHIV [60]. Myocardial fibrosis is a contributor to sudden cardiac deaths especially in PLHIV that are receiving cART [13,61]. More recently, studies have shown that persistent activation of the innate and adaptive immune responses leads to myocardial fibrosis in PLHIV (Figure 1) [32,59]. Some studies explored subclinical cardiovascular imaging changes using cardiac magnetic resonance and found that HIV-infected patients displayed changes in myocardial function and higher rates of subclinical myocardial inflammation and fibrosis, which worsened with increased severity of the disease [37].

## 5. Myocardial Fibrosis: Role in the Pathogenesis of Heart Failure and Sudden Cardiac Death

The modification of the cardiac microenvironment after injury results from the crosstalk between a variety of players such as fibroblasts, endothelial cells, inflammatory and immune cells, soluble factors, and components of the extracellular matrix (ECM) [62]. It is established that cardiac fibrosis is associated with inflammation, exemplified by continuous innate and adaptive immune responses. Myocardial fibrosis is characterized by ECM remodeling, resulting in abnormal matrix composition and leading to impairments in cardiac contractility and function. At first, ECM deposition is defensive and important for wound healing, but unnecessary or prolonged deposition can lead to impairments in tissue function. Fibrosis leads to a stiffer and less compliant heart, eventually contributing to the progression of HF and sudden cardiac death [32].

Of concern, myocardial fibrosis is emerging as a growing cardiac complication in PLHIV. For example, HIV infection (±cART) is linked to an increased incidence of myocardial fibrosis, together with systolic and diastolic LV dysfunction [61,63,64]. Some researchers found that HIV-positive patients exhibited greater evidence of myocardial fibrosis than their negative counterparts, despite relatively normal ejection fractions [32], while others showed a significantly higher prevalence of myocardial fibrosis in PLHIV who suffered mortality due to sudden cardiac deaths [65,66]. Furthermore, a study on HIV-positive patients on cART versus uninfected controls (no CVD history) found that HIV-positive patients displayed a six-fold higher rate of patchy myocardial fibrosis after controlling for age, gender, and coronary artery [61]. In addition, others evaluated associations between HIV serostatus and cardiovascular magnetic resonance imaging and demonstrated that HIV seropositivity was independently associated with greater diffuse non-ischemic fibrosis and a larger left atrial volume [67].

In terms of mechanistic insights, there is some evidence that chronic inflammation can trigger fibrosis, ECM formation, proliferation, and activation of myofibroblasts [13,62]. Activated fibroblasts and myofibroblasts are central effectors in cardiac fibrosis, by functioning as the main source of matrix proteins. Furthermore, the activation of myofibroblasts require the co-operation of growth factors and specialized matrix proteins, which signal through cell surface receptors to activate intracellular signaling pathways that can lead to the synthesis of contractile proteins and the transcription of matrix macromolecules [68]. Several cell types, such as macrophages, mast cells, and lymphocytes (infiltrating the remodeled heart), play an important role in fibroblast activation by secreting a wide range of bioactive mediators, including cytokines such as transforming growth factor (TGF)-β1 and IL-10, and matricellular proteins [62]. Furthermore, the activation of the renin-angiotensin aldosterone system stimulates fibroblast proliferation and ECM protein synthesis in the infarcted and remodeled myocardium, by activation of the angiotensin type 1 receptor or through mineralocorticoid receptor signaling [69]. Although cardiomyocyte death is usually the cause of activation of fibrogenic signals, certain stimuli such as inflammation or pressure overload may activate pro-fibrotic remodeling of the heart [62]. However, despite some progress regarding identification of the underlying mechanisms responsible for the development of myocardial fibrosis during HIV-infection, the associated risk factors and clinical consequences of such pathology still require further elucidation.

Ventricular myocytes are tightly arranged and coupled together, with adjacent layers separated by clefts [62,70,71]. Advanced proteomic methods have revealed that ~90% of the cardiac ECM comprises 10 different proteins, with serum albumin, collagens (collagens I, III, and IV), non-collagenous glycoproteins (fibronectin and laminin), proteoglycans, glucosaminoglycans, and elastins being the most common [72]. The fibrillar collagenous matrix is essentially comprised of type I (>80%) and type III (>10%) collagens [62,71,73]. Fibroblasts regulate collagen turnover by controlling the synthesis and degradation of matrix proteins [74]. As the ECM forms a link between intracellular cytoskeletal proteins and intercellular ones, this allows for the transmission of biochemical signals by mechanosensation [75]. The latter also plays a significant role in activating and differentiating myofibroblasts [75].

There are two types of myocardial fibrosis, namely reactive and replacement. Reactive fibrosis is characterized by excessive extracellular matrix deposition in interstitial or perivascular spaces and is associated with pathological conditions [62]. For example, cardiac structural abnormalities (e.g., HF, arrhythmia, and coronary artery disease) can occur due to the dysregulation of collagen metabolism (synthesis and degradation) [76]. Such structural abnormalities can cause the disruption of myocardial excitation and contraction, thereby leading to impaired systolic and diastolic function (Figure 1) [62,76]. Ventricular dysfunction is the most common cause of HF, including left-sided HF with preserved ejection fraction and reduced ejection fraction with HIV infection [77]. Excessive fibrosis can also cause mechanical stiffness, which may result in the impairment of electric conduction (forming a physical barrier between cardiomyocytes) and lead to impaired cardiac systolic function [62]. Fibrosis can also cause sliding displacement of cardiomyocytes and decrease the number of muscular layers in the ventricular wall, leading to left ventricular dilation [78]. In contrast, replacement fibrosis occurs when there is acute myocardial injury/infarction in the setting of accelerated atherosclerosis associated with HIV. This occurs due to the loss of viable myocardium and results in scar formation and LV remodeling [62,79]. Thus, a balance between replacement and reactive fibrosis is required to prevent cardiac dysfunction [78,79]. As myocardial fibrosis can elicit profound effects on myocardial function and potentially lead to HF and sudden cardiac death, understanding its pathogenesis may help identify promising targets for therapeutic interventions. For example, a recent postmortem study revealed increased rates of sudden cardiac death and myocardial fibrosis in HIV-positive persons versus non-infected individuals [65]. The contribution of myofibroblasts, monocytes/macrophages, mast cells, and lymphocytes in this context will, therefore, now be briefly discussed, although our focus is on the role of platelets in HIV-mediated cardiac fibrosis.

## 6. Monocytes/Macrophages

**Figure 2:**
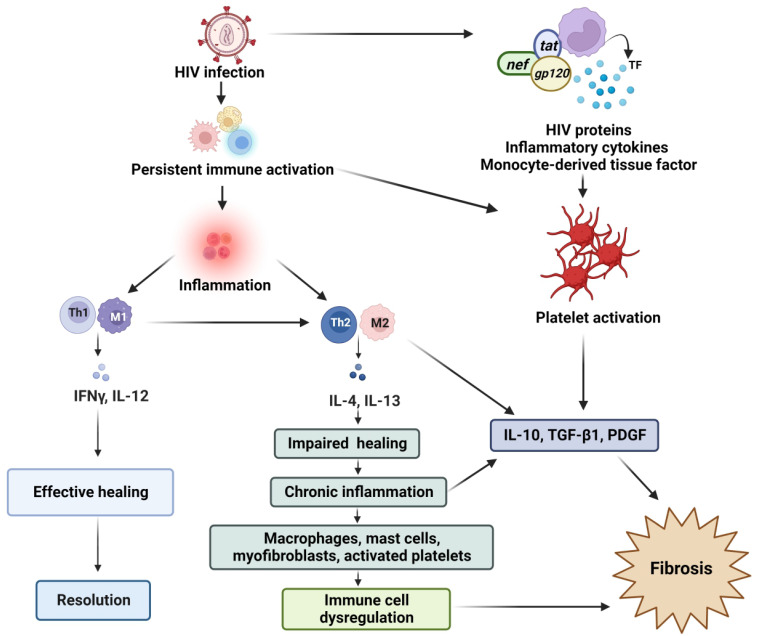
HIV infection causes the persistent activation and immune dysfunction. Effective healing is usually characterized by a dominant T helper 1 response, whereas a shift of the balance towards T helper 2 cells leads to chronic inflammation, which can eventually result in fibrosis. There are two types of macrophage activation, i.e., M1 based on the T helper 1-type response, and M2 that is based on the T helper 2-type response. The shift to T helper 2 cells, M2 responses, and platelet activation with HIV is involved in fibrotic pathway activation. HIV: human immunodeficiency virus, IFN-γ: interferon-γ, IL: interleukin, PDGF: platelet-derived growth factor, Th: T helper, TGF-β: transforming growth factor-β, TF: tissue factor.

Macrophages consist of two subsets (M1 and M2) that are implicated in cardiac remodeling [80]. M1 macrophages are pro-inflammatory and secrete inflammatory cytokines, such as IL-1 and tumor necrosis factor alpha (TNFα), whereas M2 macrophages can trigger an anti-inflammatory response. M2 macrophages play a crucial role in fibrosis by releasing pro-fibrotic mediators, such as IL-10, TGF-β, platelet-derived growth factor, and chemokines, which can recruit fibroblasts (Figure 2) [81].

Some studies found that HIV-positive women on cART displayed myocardial fibrosis (diffuse) with diastolic dysfunction [38]. Such women also exhibited increased systemic immune activation and higher sCD163 (monocyte activation marker) levels that correlated with myocardial fibrosis [38,81]. This demonstrates that monocytes can be recruited to the myocardium, propagating both myocardial inflammation and fibrosis [38,81]. Such monocytes can differentiate into macrophages, with the M2 subpopulation able to secrete anti-inflammatory cytokines and triggering collagen production by neighboring fibroblasts [38,81]. While the differentiation of M2 macrophages is associated with the progression of myocardial fibrosis [82], they can also inhibit fibrosis by phagocytosing apoptotic myofibroblasts and regulating the balance of matrix metalloproteinases and tissue inhibitors of metalloproteinases [75,81]. However, the contribution of monocytes/macrophages to the fibrotic response depends on various factors. For example, abnormal function and phenotypic changes such as the uncontrolled production of inflammatory cytokines and growth factors, an inefficient anti-inflammatory response, and/or poor communication between macrophages, fibroblasts, epithelial, and endothelial cells, can lead to aberrant repair, persistent injury, and HF [83]. Furthermore, some researchers [84] directly blocked monocyte/macrophage traffic to the heart in an SIV model of AIDS, using an anti-alpha-4 integrin antibody (natalizumab; in two groups, i.e., early and late treatment). They found decreased SIV-associated cardiac pathology in late natalizumab-treated animals compared to untreated controls. Early and late treatment resulted in significant reductions in CD163+ and CD68+ macrophages in cardiac tissues compared to untreated controls. The decreased macrophage numbers correlated with lowered fibrosis. Early and late treatment also resulted in decreased cardiomyocyte damage [84]. These data also demonstrate a role for macrophages in the development of cardiac inflammation and fibrosis, and suggest that blocking monocyte/macrophage traffic to the heart may improve HIV- and SIV-associated myocarditis and fibrosis.

## 7. Mast Cells

Mast cells are crucial participants in terms of fibrosis [75]. In support of this, increased accumulation of mast cells can contribute to cardiac remodeling and myocardial fibrosis through the release of pro-fibrotic cytokines, histamine, tryptase, and chymase [85,86]. Histamine can stimulate the proliferation of fibroblasts and collagen synthesis [62,87], e.g., the administration of a histamine H2 receptor inhibitor improved ventricular remodeling in HF patients, reflecting the pro-fibrotic effects of histamine [88]. Chymase is a protease that can enhance fibrogenic activity by elevating tissue concentrations of angiotensin II and TGF-β, both significant contributors to fibrotic signaling pathways [75]. Moreover, activated mast cells also release a wide variety of granule-stored cytokines and growth factors, such as TNFα, TGF-β, and platelet-derived growth factor, which can stimulate cardiac fibroblast proliferation and collagen synthesis [89]. However, the exact contribution of mast cells to cardiac fibrosis is still relatively unknown, as such cytokines and growth factors are also released by various other immune cells (such as macrophages and platelets).

## 8. Lymphocytes

T helper 1 cells mediate tissue damage and suppress the development of fibrosis through the release of IFN-γ and IL-12 [90]. In contrast, T helper 2 cells are pro-fibrotic through the release of IL-4 and IL-13, which are both potent stimulators of fibroblast-derived collagen synthesis. T helper 2 cells also drive macrophage differentiation towards an M2 phenotype, which further enhances the fibrotic response [62] (Figure 2). While increased expression of IL-4 and IL-13 is associated with myocardial fibrosis, the precise role of T helper 2 cells in cardiac fibrotic remodeling remains unknown [90,91]. Other T cell subpopulations are also involved in myocardial fibrosis and associated with persistent T cell-mediated inflammation [62]. For example, a large body of evidence has implicated regulatory T cells in fibrotic remodeling, especially by increased TGF-β expression and IL-10 secretion (both potent regulators of fibrosis) [92]. Moreover, T helper 17 through IL-17 generation stimulates collagen production and thereby contributes to myocardial fibrosis [75]. While the mechanisms remain to be fully elucidated, inflammatory cells appear to play an important role in myocardial fibrosis and downstream outcomes such as sudden cardiac death in PLHIV and have been reviewed elsewhere [62,65,82].

## 9. HIV-Related Myocardial Fibrosis: Role of Platelets

The contribution of platelets to myocardial fibrosis in the context of HIV remains less well understood. However, it is well established that activated platelets are an essential source of pro-fibrotic cytokines and growth factors that directly, or indirectly, stimulate a fibrotic response [93]. This can occur through the activation of fibroblasts or by promoting a fibrotic phenotype in M2 macrophages and/or lymphocytes [63]. Although persistent platelet activation is well documented in the HIV/cART setting, its contribution to myocardial fibrosis has been less emphasized [94,95]. In a systematic review and meta-analysis of 30 studies comprising 2325 participants, Nkambule et al. [96] assessed platelet activation in HIV-infected patients on cART and showed that the levels of platelet activation were elevated in treatment-naïve HIV-infected patients, persisting during treatment. While platelets contain a wide variety of pro-fibrotic cytokines and growth factors that can stimulate a fibrotic response as discussed, recent studies highlight a role for platelet-derived TGF-β in terms of myocardial fibrosis (Figure 2) [94,95].

Transforming growth factor-β is a fibrogenic growth factor that is persistently activated in animal models of cardiac remodeling and fibrosis [97,98], and that stimulates ECM protein production in different organ systems. Several preclinical studies implicate TGF-β in myocardial fibrosis: for example, Dobaczewski et al. [97] found that TGF-β1-deficient mice exhibited attenuated age-associated fibrosis [97]. In agreement, the inhibition of TGF-β prevented myocardial fibrosis in a rat model of cardiac pressure overload [99], while the genetic deletion of the TGF-β receptors in fibroblasts reduced myocardial fibrosis in an animal model of ventricular pressure overload [99]. Together, such animal-based studies highlight the role of TGF-β in cardiac fibrotic remodeling [97].

There are three known isoforms of TGF-β (TGF-β1, TGF-β2, and TGF-β3) expressed in mammalian tissues and that are encoded by distinct genes. Of note, TGF-β1 is often chronically over-expressed in fibrosis and inflammation [100]. Upon TGF-β1 release into the extracellular space it may bind to two serine-threonine kinase receptors, namely TGF-β1 receptor 1 (TβRI) and 2 (TβRII) [97]. The binding of TGF-β1 to TβRI results in the phosphorylation of Smad transcriptional modulators and the formation of a heteromeric complex that regulates DNA transcription. In the heart, the effects of TGF-β1 are mediated through Smad2 phosphorylation [101], whereafter, a complex is formed with Smad3 and Smad4. This complex translocates to the nucleus where it can bind to the regulatory regions of specific genes [101]. This complex regulates the expression of genes involved in fibrogenesis [101], including ECM proteins, such as connective tissue growth factor and periostin [101]. The increased transcription of such gene targets and others results in the production of pro-fibrotic matricellular proteins and its secretion into the ECM. This modulates intercellular and cell–matrix interactions that enhances ECM protein synthesis [102]. The TGF-β1-Smad pathways can also activate collagen-gene promoter sites, to enhance the transcription of collagen type I. An alternative pathway for TGF-β1-induced fibrosis exists and involves the TGF-β1 activated kinase (TAK1) pathway, which is activated when TGF-β1 binds to TβRII [101]. TGF-β1 activated kinase is a major downstream modulator of the TGF-β1 superfamily and is a member of the mitogen-activated protein kinase family [102]. Accordingly, the administration of TGF-β1 to cardiac fibroblasts resulted in a robust increase in TAK1 activity together with enhanced cardiac mass and significantly decreased systolic and diastolic cardiac functioning [102]. Together these studies show that targeting TGF-β signaling pathways in animal models or in clinical studies could be a novel therapeutic strategy to treat a variety of fibrotic disorders [103].

Platelets can influence plasma TGF-β levels and further enhance myocardial fibrosis. For example, plasma TGF-β levels were significantly decreased in thrombocytopenic mice with a megakaryocyte-specific deletion of the TGF-β1 gene (*Tgfb1^flox^*) following constriction of the transverse aorta. Such mice did not develop cardiac hypertrophy, fibrosis, and systolic dysfunction in response to the aortic constriction procedure [95]. The mice also survived into adulthood without abnormalities, unlike other studies where non-specific targeted deletion of the TGF-β1 gene resulted in early morbidities [95]. Moreover, the platelet counts, mean platelet volume, and function were similar to the control mice [95]. This study also suggests possible therapeutic interventions to explore, especially within the clinical context.

Others found that fibrosis can be linked to platelet activation and TGF-β1 release [93]. For example, investigators treated mice with daily, pharmacological doses of ritonavir (potent HIV protease inhibitor) for 8 weeks [93]. Here, mice with a targeted TGF-β deletion in megakaryocytes were partially protected from ritonavir-induced cardiac dysfunction and fibrosis versus the controls [93]. The fibrosis correlated with plasma TGF-β levels and the activation of the Smad2/3 and TAK1/MKK3/p38 pathways in the heart [93]. The significant contribution of platelet-derived TGF-β to myocardial fibrosis may be due to its relatively high expression in platelets (40–100× more than other cells) and its rapid release upon activation [104].

With HIV-infection there are three mechanisms of platelet activation that can result in TGF-β release, i.e., (a) binding of the HIV viral envelope to dendritic cell-specific intercellular adhesion molecule-grabbing non-integrin (pathogen receptor expressed on platelets) [63,105], (b) stimulation by inflammatory cytokines (IL-6, IL-8, and IL-1β) [63,105], and (c) thrombin generation mediated via monocyte-derived tissue factor, which is significantly increased in HIV-positive patients (both in its soluble state and expression on monocytes) [106]. Furthermore, certain cART types (i.e., HIV protease inhibitors) can also promote platelet activation [107]. Thus, platelet activation can persist and induce the secretion of platelet-derived TGF-β [63], to thereby result in myocardial fibrosis.

While platelets contribute to ~80% of TGF-β, in terms of the development of cardiac fibrosis [63], HIV-related fibrosis is multi-factorial and other inflammatory cells can further exacerbate such a pathology. For example, endothelial cell injury, together with activated monocytes/macrophages and platelets, can lead to the production of reactive oxygen species and oxidative stress [94]. Reactive oxygen species is a potent activator of TGF-β, and its generation occurs relatively early-on during HIV infection, despite effective cART [94]. This creates a positive feedback loop with platelet activation and the transition of latent TGF-β to its active, pro-fibrotic form [94]. Moreover, HIV protease inhibitors (e.g., ritonavir) can further exacerbate platelet activation and pro-fibrotic signaling. Such deleterious effects may be directly mediated and/or may occur indirectly through the induction of oxidative stress [63,93,107,108].

## 10. Conclusions

**Figure 3:**
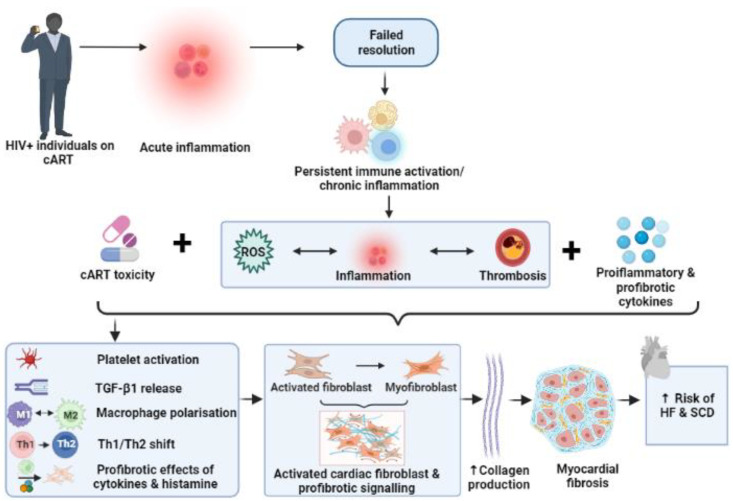
The inflammatory hypothesis and cardiac fibrosis. Failed resolution during early HIV infection leads to persistent immune activation and a chronic inflammatory status, together with thrombosis, increased levels of ROS and proinflammatory and profibrotic cytokines. Immune dysregulation (e.g., platelet activation, macrophage polarization, and shift of T helper cells) results in increased fibroblast activation and profibrotic signaling, which leads to myocardial fibrosis. The culmination of such factors increases the risk for heart failure and sudden cardiac death. ROS: reactive oxygen species, cART: combination antiretroviral therapy, HIV: human immunodeficiency virus, HF: heart failure, SCD: sudden cardiac death, Th: T helper, TGF-β: transforming growth factor-β.

Fibrosis is a physiologic response to the physical, chemical, and biologic injuries that are associated with inflammation. However, HIV-related persistent immune activation exhibits relatively high levels of inflammatory and pro-fibrotic cytokines, the generation of reactive oxygen species, the activation of latent TGF -β1, as well as polarization of macrophages and T helper cell shifts. Here, pathologic tissue fibrosis can result in permanent scarring and cardiac dysfunction in HIV-positive patients, leading to HF and sudden cardiac death (Figure 3). The current diagnosis of cardiac fibrosis is hampered by the invasive and expensive nature of gold standard techniques, such as endomyocardial biopsies and cardiac magnetic resonance imaging, respectively. Surrogate markers of fibrosis include biomarkers and histochemical staining, which are inadequate to fully characterize such pathology. Hence, future studies should investigate more comprehensive, non-invasive, and cost-effective techniques, which can be routinely used for health checks, especially in the developing world context. As HIV-mediated CVD is a major global burden of disease (especially in resource-poor regions), additional studies focusing on anti-inflammatory, as well as anti-fibrotic, therapeutic interventions may help to improve and/or counter this growing clinical problem. Furthermore, preventive cardiovascular care should not be neglected in preference to the control of viremia in PLHIV, to ensure optimal management of the CVD burden in such individuals.

## Data Availability

Not applicable.
